# A Pilot Study to Detect Viable *Salmonella* spp. in Diarrheal Stool Using Viability Real-Time PCR as a Culture-Independent Diagnostic Tool in a Clinical Setting

**DOI:** 10.3390/ijms24129979

**Published:** 2023-06-10

**Authors:** Surangi H. Thilakarathna, Linda Chui

**Affiliations:** 1 Department of Laboratory Medicine and Pathology, University of Alberta, Edmonton, AB T6G 1C9, Canada; sthilaka@ualberta.ca; 2 Alberta Precision Laboratories, Public Health Laboratory (ProvLab), Edmonton, AB T6G 2J2, Canada

**Keywords:** viability assessment, qPCR, viability qPCR, false positives, diarrheal stools, stool enrichment, *Salmonella* spp., clinical setting

## Abstract

Frontline laboratories are adopting culture-independent diagnostic testing (CIDT) such as nucleic acid amplification tests (NAATs) due to numerous advantages over culture-based testing methods. Paradoxically, the viability of pathogens, a crucial factor determining active infections, cannot be confirmed with current NAATs alone. A recent development of viability PCR (vPCR) was introduced to mitigate this limitation associated with real-time PCR (qPCR) by using a DNA-intercalating dye to remove residual and dead cell DNA. This study assessed the applicability of the vPCR assay on diarrheal stools. Eighty-five diarrheal stools confirmed for Salmonellosis were tested via qPCR and vPCR using in-house primers and probe targeting the *invA* gene. vPCR-negative stools (Ct cut off > 31) were enriched in mannitol selenite broth (MSB) to verify low bacterial loads. vPCR assay showed ~89% sensitivity (qPCR- and vPCR-positive stools: 76/85). vPCR-negative stools (9/85; qPCR-positive: 5; qPCR-negative: 4) were qPCR- and culture-positive post-MSB-enrichment and confirmed the presence of low viable bacterial loads. Random sampling error, low bacterial loads, and receiving stools in batches could contribute to false negatives. This is a pilot study and further investigations are warranted to explore vPCR to assess pathogen viability in a clinical setting, especially when culture-based testing is unavailable.

## 1. Introduction

Acute gastroenteritis continues to be a major public health concern as of today. Around 4 million Canadians are infected with food-borne enteritis each year, leading to ~11,600 hospitalizations and ~240 deaths [1]. The accurate and timely diagnosis of the pathogen is crucial for treatment and patient care. Until recently, culture-based diagnostic tests were the gold standard for pathogen detection and identification. In recent years, most frontline microbiology diagnostic laboratories have adopted culture-independent diagnostic testing (CIDT) using nucleic acid amplification tests (NAATs) [2,3,4]. NAATs such as real-time PCR (qPCR) are designed for automation, multi-analyte, high-volume testing, and have a rapid turnaround time for reporting [3,4,5]. Given these and many other advantages, frontline laboratories tend to report enteric infections based on NAAT results alone without culture confirmation [2,4,5,6]. This can be problematic due to a significant limitation associated with NAATs: the lack of selectivity between the amplified product from “live” and “dead” cells. Therefore, a positive NAAT result may not necessarily indicate an active infection.

Recently, there has been a development of viability qPCR (vPCR) that can selectively amplify live cell DNA in samples consisting of a mixture of live and dead cells [7,8]. vPCR assay uses bacterial cell membrane integrity to assess cell viability by using a DNA intercalating dye, i.e., propidium monoazide (PMA) or ethidium monoazide (EMA) [7,9,10,11]. These viability dyes can penetrate through compromised cell membranes and irreversibly bind their DNA [7,8]. These viability dyes are believed to be impermeable to live cells with intact cell membranes; however, there are studies that report EMA uptake by live cells [7,12,13]. The impermeability of PMA to live cell membranes was shown to be superior to EMA [7]. Therefore, by coupling a viability dye treatment such as PMA, a qPCR assay can potentially be upgraded to a NAAT that assesses viability.

The applicability of vPCR assay to different matrices has been previously tested. As an example, PMA dye treatment was tested on suspensions of pure bacterial broth cultures [11,14,15], wastewater treatment plant influents and effluents [16], artificially contaminated food [17,18], urine [19], and piggery effluents [20]. A few studies have tested the applicability of PMA dye treatment on complex, heterogenous matrices such as stools and biosolids, i.e., on human [21,22,23] and equine [24] donor fecal slurries prepared for transplantation to assess the viability of the donor microbiome.

In our previous work, we reported the optimization of a vPCR assay using PMAxx™ (an improved version of PMA) as the intercalating dye and pure culture of *Salmonella enterica* subsp. *enterica* ser. Enteritidis (*S*. Enteritidis) as the target organism [15]. As the next phase of our work, we sought to investigate the applicability of the vPCR assay in a clinical setting. As a pilot, this study evaluated the applicability of the vPCR assay using PMAxx™ as the DNA intercalating dye to assess the viability of *Salmonella* spp. in diarrheal stool specimens in parallel with qPCR and culture. To the best of our understanding, this is the first study that has tested the vPCR assay on clinical diarrheal stools.

## 2. Results

### 2.1. Standard Curve Generated from Bacteria Cells Spiked in Salmonella—Negative Stool Broth

**Figure 1:**
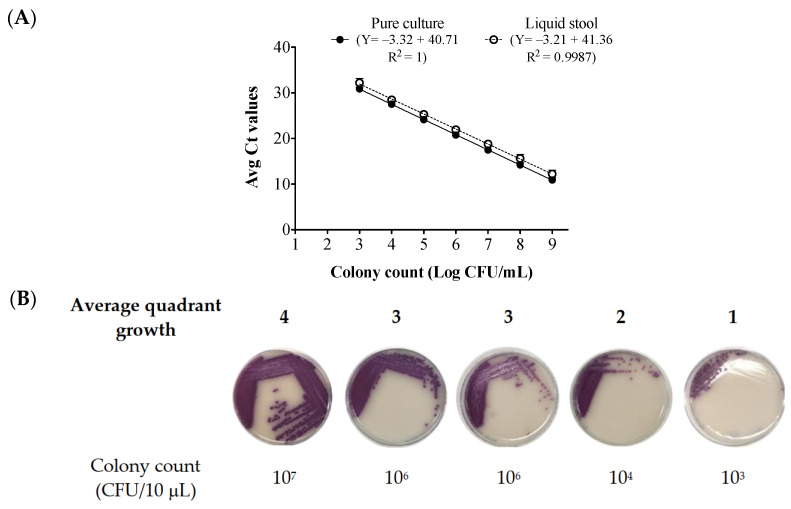
Correlation between the average Ct values, the cell counts, and the quadrant growth of *Salmonella enterica* subsp. *enterica* ser. Enteritidis in pure culture and in spiked *Salmonella*-negative stool broth. (**A**) Standard curve between average Ct values and average colony counts (CFU/mL) for pure culture and *S*. Enteritidis-spiked *Salmonella*-negative stool broth. Data were from 18 replicates of pure culture and 18 replicates of spiked stool broth with triplicates per qPCR run. Data are displayed as mean ± SD. Colony counts were obtained via growth in culture. (**B**) The average quadrant growth on CHROMagar™ Salmonella Plus plates (n = 18). The displayed images of quadrant growth on plates are not from the same replicate run and were selected to best represent the average quadrant growth of the 18 spiked stool broth replicates. Scoring on plates was conducted in triplicate for each spiked stool broth replicate. Ten microliters from each dilution was used for plating. Detection limit for culture growth on CHROMagar™ Salmonella Plus plates: 10^3^ CFU/10 μL. Ct: cycle threshold, CFUs: colony-forming units.

The stool broth standard curve was constructed using average cycle threshold (Ct) values and colony counts in log-colony-forming units (CFUs)/mL obtained from dilutions of *S*. Enteritidis cells spiked in individual *Salmonella*-negative stool replicates (n = 18). A standard curve from the corresponding dilutions of the pure culture (n = 18) was developed in parallel (Figure 1A).

**Figure 2:**
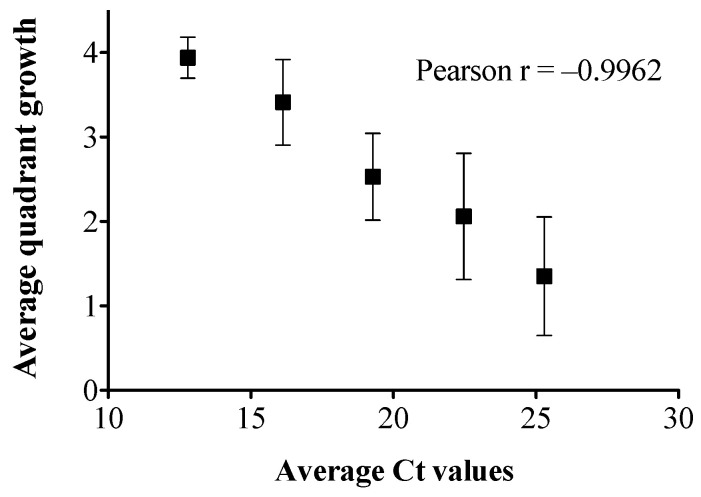
Correlation between the average Ct values and average quadrant growth of the liquid stool spiked with different *S*. Enteritidis cell dilutions.

We previously reported that the Ct cut off value for the in-house qPCR assay was at 31 [15]. The growth of the 10^−6^ and 10^−7^ dilutions of the pure culture on blood agar plates (BAP) and CHROMagar™ Salmonella Plus plates confirmed the original cell concentration to be 10^9^ CFU/mL. There was a significant Ct difference of 0.9 to 1.4 between different dilutions of pure culture and culture-spiked liquid stool broth standard curves (*p* < 0.05, Figure 1A). The results of the quadrant growth of each spiked stool broth dilution on CHROMagar™ Salmonella plus plates is shown in Figure 1B. The stool broth quadrant growth and the average Ct values were inversely correlated (*p* < 0.05, Pearson r = –0.9962, Figure 2).

### 2.2. Clinical Stool Analysis

A total of 85 stool aliquots were analyzed via qPCR and vPCR (Supplementary Table S1). These stools were liquid or semi solid in consistency and were categorized under type 6 and 7 according to the Bristol Stool Form Scale [25]. All stools were from patients suffering from acute gastroenteritis and were screened as being positive for *Salmonella* spp. via BD MAX™ followed by culture in the primary microbiology laboratory. The isolates were submitted to the Alberta Precision Laboratories, Public Health Laboratory (ProvLab) for molecular subtyping. The serotypes of the *Salmonella* spp. identified in this study were *S*. Enteritidis (n = 40), *S.* Typhimurium (n = 8), *S.* Muenchen (n = 1), *S.* Newport (n = 5), *S.* Paratyphi B var Java (n = 2), *S.* Goldcoast (n = 1), *S.* Infantis (n = 2), *S.* Uganda (n = 2), *S.* Reading (n = 1), *S.* Braenderup (n = 4), *S.* Kiambu (n = 2), *S.* Javiana (n = 1), *S.* Thompson (n = 1), *S.* Anatum (n = 1), *S.* Indiana (n = 2), *S.* Heidelberg (n = 1), *S.* Weltevereden (n = 1), *S.* Livingstone (n = 1), *S.* Bareilly (n = 1), *S.* Senftenberg (n = 1), *S. enterica* subsp. Diarizonae (IIIb) 61:z52:z53 (n = 1), *S. enterica* subsp. *enterica* (I) O Rough:z4,z23: (n = 1), *S. enterica* subsp. *enterica* (I) 4,[5],12:i:- (n = 1), *S.* St Paul (n = 1), *S.* Munster (n = 1), *S.* Corvallis (n = 1), and *S.* Kentucky (n = 1).

**Figure 3:**
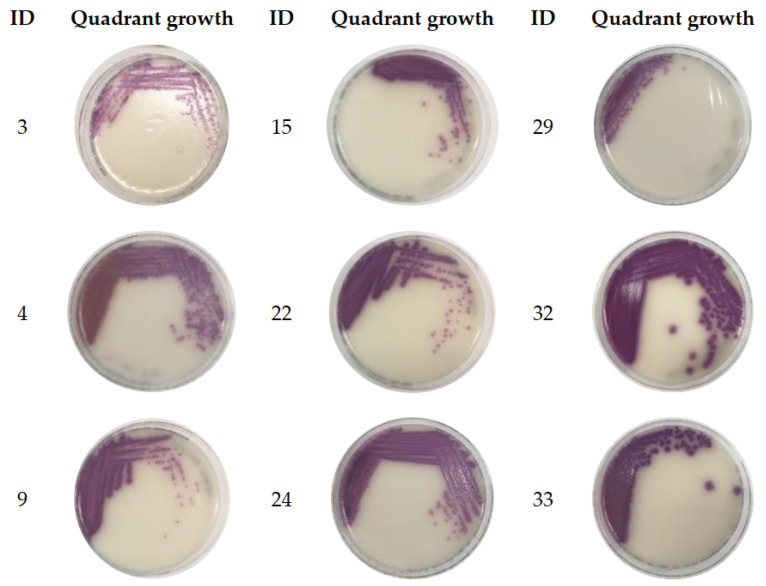
Post-enrichment quadrant growth for stool samples with negative vPCR (n = 9). A 10 μL aliquot of each enriched stool broth was separately plated in quadrants on CHROMagar™ Salmonella Plus plates and was incubated overnight.

No qPCR inhibition was observed in the amplification of neat (undiluted) or a 1/10 dilution of DNA extracted from spiked culture in stool broth samples (Supplementary Table S1). From the stools analyzed, 76/85 (89%) showed positive qPCR and vPCR results (Supplementary Table S1), pointing to a vPCR assay sensitivity of ~89%. Twenty-four of these samples had a Ct difference (ΔCt) of < 1 between qPCR and vPCR results. For 14/76 stools, the ΔCt for vPCR-qPCR was > 3. Nine stools tested negative for both qPCR and vPCR (vPCR and qPCR Ct ≥ 31), where 4/9 were qPCR negative. All vPCR-negative stools (n = 9) were enriched in Mannitol Selenite Broth (MSB) overnight to verify the presence of low viable bacterial loads. qPCR analysis on aliquots of the post-enriched stool broths showed a significant Ct reduction (*p* < 0.05) compared to pre-enrichment with CT values ranging between ~11 and 18. The ΔCt between pre-enrichment vPCR and post-enrichment qPCR was > 17 Ct values (Table 1). Furthermore, the viability of the bacteria post-enrichment was confirmed via the growth of 3–4 quadrants of mauve colored colonies on CHROMagar™ Salmonella Plus plates, except for ID 29 (Figure 3). The growth of only one quadrant for the enriched ID 29 stool was an unanticipated observation.

### 2.3. Colony Count Estimation in Enriched Clinical Stools Using the Stool Broth Standard Curve

Based on the standard curve generated using different dilutions of bacterial cells spiked into stool broths (Figure 1A), the Ct values obtained for the post-enrichment qPCR (Table 1) can roughly predict the range of bacterial load in the enriched MSB. Accordingly, the enriched stool broths consisted of ~10^8^–10^9^ CFU/mL based on the standard curve (Figure 1A) and the observed quadrant growth was 3–4. According to the qPCR readings and the correlation between the average quadrant growth and average Ct values (Figure 2), 3–4 quadrants growth was predicted when Ct values < 16.

## 3. Discussion

This is a pilot study that explored the possibility of applying the vPCR assay in a clinical setting. The viability of *Salmonella* spp. in stools submitted by patients experiencing diarrhea was tested by performing qPCR and vPCR assays in parallel. The qPCR-positive results reflected the presence of any *Salmonella* DNA in the stools (from either dead or live cells or residual DNA) while vPCR-positive results reflected the presence of DNA from only viable *Salmonella* cells [7,11,26]. The stools that showed a ΔCt of < 1 between the two PCR assays indicated the presence of more live cell DNA than dead cell and/or free DNA. The increase in ΔCt between qPCR and vPCR (qPCR Ct < vPCR Ct) could be an indication of having more dead cells and/or residual DNA in stools compared to live cell DNA. Hence, the stools that showed > 3 Ct difference between qPCR and vPCR possibly had ~ 1 log CFU/mL more dead cell DNA than live cell DNA.

All stools were initially confirmed for *Salmonella* positivity via BD MAX™ and culture with isolates serotyped. This information was crucial to confirm that the vPCR-negative results were in fact false negatives caused by low bacterial loads and not true negatives due to the absence of *Salmonella*. Based on our data, the sensitivity of the vPCR assay was ~89%, where 76/85 stools were true *Salmonella* positives while 9/85 were false negatives (Sensitivity of an assay: True positives/ (True positives + False negatives) × 100) [27]. Many factors could have contributed to the false vPCR negatives: different sensitivities of the primers used in the BD MAX™ and the in-house PCR assay, the presence of low bacterial loads that were below the detection limit of the assay, interference of PMAxx™ on viable cells, etc. BD MAX™ (*spaO* gene; BD MAX™ Enteric Bacterial Panel information sheet) and the in-house qPCR assay (*invA* gene; [15]) used different gene targets; however, the in-house qPCR assay (10^3^ CFU/mL [15]) was more sensitive than the BD MAX™ assay (10^5^ CFU/mL [28]). So, the differences in the sensitivity of the primers can be considered less as a contributing factor to the detection of vPCR false negatives. The loss in cell viability can be a contributor as the stool samples were received in batches for this study. Therefore, it is possible that when the initial bacterial cell count was low in the submitted stool, the viability was lost due to the lag of time between the BD MAX analysis and receipt of the stool samples for PCR and vPCR analysis. The interference of stool constituents on PMAxx™ activity can be another possible contributor that reduced vPCR assay sensitivity. A decrease in the qPCR Ct values and culture positivity post-MSB-enrichment provided the evidence of low viable bacterial loads in the initial submitted samples at a concentration below the detection limit of our vPCR assay. As previously reported, the level of detection for the in-house qPCR assay was at 100%, ~76%, and ~3%, corresponding to 1000 (Ct~31), 100 (Ct~34), and 10 (Ct ~36) CFU/mL, respectively [15]. With PMAxx™ treatment, the sensitivity of the subsequent qPCR was further reduced from 10^3^ to 10^4^ CFU/mL (~ 30 Ct corresponding to 10^4^ and Ct undetected for 10^3^ CFU/mL [15]). Therefore, viable bacterial cells below 10^4^ CFU/mL might not be 100% detected from our vPCR assay. The effects of stool constituents on vPCR assay sensitivity were not tested in this study.

We reported in our previous work that the false negatives were not eliminated from the optimized vPCR assay, and as a result, the assay sensitivity was reduced [15]. These findings were in agreement with previous studies on pure bacterial cultures [14,29]. When using stool suspensions or complex sample matrices such as biosolids and effluents (i.e., piggery effluents), inherent sample characteristics such as heterogeneous nature, larger solid particles, turbidity of the sample, etc. can also affect assay sensitivity [16,20]. Studies have reported that the total solids present in the stool [16], the turbidity of the stool or stool suspension [20,21], and the presence of organic matter caused a decline in the vPCR assay sensitivity. Photoactivation is crucial for PMAxx™ activity, and therefore hindered light penetration due to sample turbidity can significantly diminish PMAxx effectiveness [21]. PMAxx™ has a tendency to bind to surfaces other than DNA even post-photoactivation [15], and consequently, it will be unavailable to bind to the dead cell DNA and free DNA once bound to solid particles in stools. Because of this, direct stool was not recommended for vPCR analysis due to the high amounts of solids present. Sample dilution appeared to be the most effective solution for this problem and it is crucial to select the most appropriate level of dilution to avoid over-dilution [16,20,21]. Therefore, caution should be exercised when deciding on the dilution factor. Since we used diarrheal stools, the water content of the stools was much higher and solids were much lower than the normal stools. Furthermore, the larger stool particles were let to settle prior to aliquoting the 10% stool suspension for qPCR and vPCR analysis to minimize the interference from stool particles on PMAxx™ activity. Unlike pure bacterial cultures, stools can consist of residual and dead cell DNA from not only the target bacteria of interest but also other numerous sources. Since PMA and PMAxx™ have no selectivity toward binding to DNA from a specific species or strain, the dye treatment should be sufficient enough to bind to all dead cell and residual DNA in the stool sample in order to ensure the removal of dead cell and residual DNA from the target of concern. Using a higher concentration of the dye treatment, i.e., 100 μM, and sample dilution (diluting the available amount of dead cell and residual DNA) would be the most practical solutions.

Ideally, to test the effectiveness of the viability dye treatment, an alternate secondary assay is necessary to confirm the observed vPCR results. Finding an accurate yet practical secondary assay to validate vPCR results was challenging. The most feasible method we came across was culturing the stools in selective media for *Salmonella* spp. However, the culture growth on CHROMagar™ Salmonella plus plates did not always agree with the vPCR results, pointing to the impact of stool microflora on culture-based assay sensitivities [30]. In our study, the growth of *Salmonella* spp. spiked into individual stool broth replicates showed vast differences in the mauve colonies observed on chromogenic agar media (Supplementary Figure S1). These observations were only possible as individual stool replicates were used instead of pooled stool replicates. Some stool microflora masked the growth of *Salmonella* spp. resulting in fine mauve colonies, where some stool environments promoted the growth of *Salmonella* spp. resulting in distinct large mauve colonies. This confounding effect of stool microflora and stool particulate on assay sensitivities as discussed above can be problematic, especially when these secondary assays are used for the purpose of confirming vPCR assay results.

In the province of Alberta, Canada, stool specimens submitted by patients presenting gastroenteritis symptoms are screened using BD MAX™, a commercial multi-analyte, real-time PCR platform for detecting enteric pathogens. All positive CIDT results are to be confirmed by culture, so that isolates can be further characterized for serotyping, cluster analysis, outbreak detection, and surveillance [3]. However, there is an emerging trend that frontline laboratories consider only NAATs for pathogen detection with no follow up confirmation by culture [2,4,5]. This can be problematic as these results might not always be an indication of an active infection [31]. In such cases, vPCR can be immensely beneficial as a viability assessment tool when confirmation by culture is unavailable or practically not possible. NAAT-positive and culture-negative stool specimens are recommended to be considered as probable cases during initial diagnosis and a repeat culture is suggested for the confirmation of pathogen viability [3]. Especially in such situations, a vPCR assay can be used to confirm the viability of the pathogen. This can significantly reduce the time taken for case confirmation. As a matter of fact, with further improvements by using better gene targets and more sensitive fluorescent probes, it will be possible to have vPCR replacing qPCR in the future as a screening diagnostic tool because of its ability to predict the viability of the pathogen.

## 4. Materials and Methods

### 4.1. Standard Curve Construction

#### 4.1.1. Bacterial Strains and Growth Conditions

*Salmonella* Enteritidis was used for constructing the standard curves correlating the Ct values of qPCR and CFU of the bacteria. *S*. Enteritidis was retrieved from frozen skimmed milk stored at −80 °C, and was cultured on BAP (Dalynn Biologicals, Calgary, AB, Canada) and incubated at 37 °C overnight. On the following day, a single colony was picked, inoculated into 4 mL of trypticase soy broth (TSB, Dalynn Biologicals, Calgary, AB, Canada), and incubated at 37 °C with moderate shaking (Model 4365, Thermo Fisher Scientific, Oakwood, OH, USA) for around 3.5 h until the OD value reached ~0.5 (MicroScan Turbidity Meter, Siemens Healthcare Diagnostics Ltd., Los Angeles, CA, USA). A ten-fold serial dilution in phosphate saline buffer (PBS, Gibco™, Life Technologies, Grand Island, NY, USA) from neat to 10^−9^ was prepared with this standardized cell suspension and used in the stool spiking experiments. One hundred microliters of the 10^−6^ and 10^−7^ dilutions were inoculated onto BAP in triplicates and incubated overnight at 37 °C to estimate the bacterial cell concentration (CFU/mL) of the standardized cell suspension.

#### 4.1.2. Stool Spiking with *S*. Enteritidis

The standard curves were developed by performing the below experiments to simulate the growing conditions of the stool containing *Salmonella* spp. in broth. Diarrheal stools that initially tested negative for *Salmonella* spp. were collected from Alberta Precision Laboratories, Public Health Laboratory (ProvLab) and the *Salmonella* negativity was re-confirmed using an in-house qPCR assay targeting the *invA* gene [15]. All of these stools were categorized under “Type 7” (liquid consistency with no solid pieces) according to the Bristol Stool Form Scale [25]. A total of 300 μL of *Salmonella*-negative stools were inoculated into 9 mL of MSB (Dalynn Biologicals, Calgary, AB, Canada) and incubated at 37 °C overnight. On the following day, the incubated stool broth was thoroughly mixed and large particles were let to settle at room temperature prior to using the supernatant for spiking experiments. A ten-fold serial dilution of *S*. Enteritidis was prepared as described in Section 4.1.1., and 500 μL from each cell dilution in PBS was pipetted into separate microcentrifuge tubes which were subjected to centrifugation at 17,115× *g* for 5 min. The PBS supernatant was then removed from each dilution and 500 μL of the stool broth supernatant was added and mixed well to disperse the cell pellet. An aliquot of the spiked stool broth was used for nucleic acid extraction, as described below.

### 4.2. Nucleic Acid Extraction

A 100 μL aliquot of the spiked stool broth with each dilution of cell suspension was centrifuged at 17,115× *g* for 5 min. The supernatant was removed, and the cell pellet was resuspended in 100 μL of rapid lysis buffer (RLB, 100 mM NaCl, 10 mM Tris-HCL pH 8.3, 1 mM EDTA pH 9.0, 1% Triton X-100) and boiled at 95 °C for 15 min. The suspension was centrifuged at 9391× *g* for 5 min and 5 μL of the supernatant was used as the template for qPCR.

### 4.3. qPCR and vPCR Assay Conditions

#### qPCR Assay

The in-house primer–probe (Integrated DNA Technologies, IDT, Skokie, IL, USA) set that targeted a conserved region of the *Salmonella invA* gene [15,32] was used in the qPCR assays. The primer and probe sequences, their sensitivity, and the specificity have been previously reported [15]. The qPCR reaction mixture was prepared by combining 10 μL of 2X PrimeTime^®^ Gene Expression Master mix (Integrated DNA Technologies, IDT, Skokie, IL, USA), 2 μL of nuclease-free water (Invitrogen™, Live Technologies, Grand Island, NY, USA), 3 μL of the in-house primer–probe mixture (0.22 μM final concentration of the probe, 0.33 μM final concentration of each of the primers), and 5 μL of the DNA template in a final reaction volume of 20 μL. Positive DNA for the *invA* gene and a no-template control (nuclease-free water) were included in each run. Amplification conditions consisted of 95 °C for 3 min followed by 40 cycles of 95 °C for 5 s and 60 °C for 30 s using the 7500 FAST real-time PCR system (Applied Biosystems, Foster City, CA, USA).

### 4.4. Clinical Stool Analysis

#### 4.4.1. Clinical Stool Samples

Aliquots of patient diarrheal stool samples that tested positive for Salmonellosis using BD MAX™ at the primary microbiology diagnostic laboratory were collected between April 2022 and January 2023. These specimens were shipped to our laboratory in batches and were stored at 4 °C upon receipt. qPCR and vPCR assays were performed within 14 days of initial stool collection for this investigation. The consistency of each stool according to the Bristol Stool Form Scale [25] was recorded.

#### 4.4.2. PMAxx™ Preparation for vPCR Assay

PMAxx™ was used as the intercalating dye for the vPCR assay at a final concentration of 100 μM. To achieve this final concentration, a 20 mM PMAxx™ solution in water (Biotium Inc., Fremont, CA, USA) was diluted with dimethylsulfoxide (DMSO, Sigma-Aldrich, St. Louis, MO, USA) and sterile DNA/RNA free water (Invitrogen™, Life Technologies, Grand Island, NY, USA) to prepare a 2 mM PMAxx™ stock solution. The concentration of DMSO in the 2 mM PMAxx™ stock solution was 20% and in the final reaction mixture it was 1%. The PMAxx™ stock solution was stored at −20 °C in the dark until it was used. To preserve the activity of PMAxx™, experiments were performed under minimal lighting conditions.

#### 4.4.3. qPCR and Viability Assessment via vPCR and Plate Growth

For *Salmonella*-positive stools submitted, a 10% (*w*/*v*) stool suspension was prepared by weighing the appropriate amount of stool and suspending it in PBS. The stool suspension was then thoroughly mixed via vortexing for ~30 s and was allowed to settle for ~30 min. A total of 100 μL of the stool supernatant was centrifuged at 17,115× *g* for 5 min and the supernatant was removed. Then, the pellet was resuspended in RLB and nucleic acids were extracted, as in Section 4.2. For PMAxx™ treatment, 5 μL of 2 mM PMAxx™ reagent was added to a total of 100 μL of the stool supernatant. After vortexing briefly, the stool suspension was incubated at room temperature for 10 min in the dark followed by exposure to intense visible light using the PMA Lite™ LED photolysis device (Biotium Inc., Fremont, CA, USA) for 15 min at room temperature. After the photoactivation period, the suspension was centrifuged at 17,115× *g* for 5 min and the supernatant was removed. The pellet was then resuspended in RLB and the nucleic acids were extracted, as in Section 4.2. Both untreated and PMAxx™-treated nucleic acid extracts were tested using qPCR. A 1:10 dilution of untreated and PMAxx™-treated cell lysates were also analyzed via qPCR to ensure there was no PCR inhibition.

#### 4.4.4. Enrichment of Stools with Low Bacterial Loads in Mannitol Selenite Broth

A vPCR Ct cut off value > 31 from the stool samples was selected for enrichment in MSB. A pea-sized amount of semi solid stool or 100 μL of liquid stool was inoculated into 3 mL MSB and incubated overnight at 37 °C. A color change in the incubated stool broth from colorless to bright red was indicative of the possible growth of *Salmonella* spp. qPCR assay was performed on 100 μL of the DNA extracted from the enriched broth on the following day, and 10 μL of the enriched broth was streaked into 4 quadrants on CHROMagar™ Salmonella Plus plates and incubated at 37 °C for at least 20 h to estimate the bacterial load in the broth. The presence of mauve colored colonies in quadrants on CHROMagar™ plates after incubation was considered as positive growth.

### 4.5. Statistical Analysis

Statistical analysis was performed using GraphPad Prism statistical software version 5 (GraphPad Software, Inc., San Diego, CA, USA). Analysis of variance (ANOVA) was used to compare different cell dilutions of pure culture and spiked stool broth. Bonferroni post-test was used to analyze treatment differences, and the differences were considered statistically significant when *p* < 0.05. Pearson correlation analysis was performed between the average quadrant growth and the average Ct values of the *S*. Enteritidis-spiked stool.

## 5. Conclusions

A viability qPCR assay using PMAxx™ as the viability dye was tested on diarrheal stools that initially tested positive for Salmonellosis. For the diarrheal stools tested, the vPCR assay showed ~89% sensitivity. Around 11% false negatives (n = 9) were detected and 4/9 of these false negatives were both qPCR- and vPCR-negative. These four samples pointed to *Salmonella* cells/DNA below the detection limit of both qPCR and vPCR assays. Enrichment in a selective broth, i.e., MSB confirmed that, in fact, the false negatives were caused by the low bacterial loads. The presence of live pathogen cells even at very low concentrations can be problematic in a clinical setting. Therefore, the findings from this pilot study emphasized the importance of assay optimization in order to minimize false negatives. In this regard, controlled stool spiking studies using both live and dead bacterial cells are required to confirm the effectiveness of the PMAxx™ treatment and to study the bacterial cell concentration at which false positives and false negatives can be potentially generated. In general, the tendency of the vPCR assay to produce false negative results warrants further investigations. There is a need to explore approaches to further improve the vPCR assay: better gene targets, more sensitive fluorescent probes, better-performing viability dyes/compounds, etc. With improved diagnostic sensitivity and further testing on other Gram-negative and Gram-positive gastroenteric bacterial pathogens, vPCR has great potential to be applied as a multiplex method to subsequently assess the viability of multiple bacterial pathogens.

## Figures and Tables

**Table 1:** Pre- and post-enrichment data for vPCR-negative stool samples (n = 9).

ID	Pre-Enrichment Data			Post-Enrichment Data	
	qPCR (Ct Value)	vPCR (Ct Value)	Stool Culture	qPCR (Ct Value)	Quadrant Growth
3	34.48	36.34	POS	13.76	3
4	29.71	32.67	NEG ^#^	16.20	3
9	30.53	33.08	POS	12.12	3
15	30.43	34.69	POS	12.16	3
22	31.36	33.73	POS	13.01	3
24	29.88	31.92	POS	12.15	4
29	27.39	30.70	POS	12.34	1
32	31.82	31.88	POS	12.53	4
33	UD	32.25	POS	13.20	3

Pre-enrichment qPCR and vPCR was performed on the same day. Stools were cultured on CHROMagar™ Salmonella plus plates within 3 weeks of initial collection. ^#^ Culturing on plate was performed > 30 days after initial stool collection. Appearance of mauve colored colonies after incubation indicated culture positivity. Ct: cycle threshold; UD: undetected; POS: positive, NEG: negative.

## Data Availability

Not applicable.

## Supplementary Materials

The following supporting information can be downloaded at https://www.mdpi.com/article/10.3390/ijms24129979/s1.
