# Supplementary material for: A Pilot Study to Detect Viable Salmonella spp. in Diarrheal Stool Using Viability Real-Time PCR as a Culture-Independent Diagnostic Tool in a Clinical Setting
Source: Int J Mol Sci. 2023 Jun 10;24(12):9979. doi: 10.3390/ijms24129979 (PMC10298261; doi:10.3390/ijms24129979)
Supplement: Supplementary file 1 [file ijms-24-09979-s001.zip › ijms-2426255-supplementary.pdf]

Supplementary Table S1. qPCR and vPCR data of the clinical stools tested

ID	Average Ct value				ID	Average Ct value			
	qPCR		vPCR			qPCR		vPCR	
	Neat	1:10 dilution	Neat	1:10 dilution		Neat	1:10 dilution	Neat	1:10 dilution
1	22.10	25.15	22.84	26.54	44	25.92	NA	27.93	NA
2	15.87	18.84	16.75	20.60	45	17.55	NA	18.05	NA
**3**[*]	**34.48**	36.47	**36.34**	35.36	46	21.98	NA	22.82	NA
**4**[#]	**29.71**	33.28	**32.67**	35.93	47	17.21	20.27	17.92	19.49
5	17.67	20.75	18.53	21.67	48	21.46	25.03	23.15	26.77
6	18.77	21.81	20.01	23.26	49	28.46	32.74	29.56	32.95
7	16.89	19.84	18.89	22.44	50	25.65	28.63	26.20	30.10
8	16.32	19.74	18.47	21.66	51	16.11	19.25	17.01	20.32
**9**[#]	**30.53**	UD	**33.08**	34.04	52	27.27	30.49	29.02	31.93
10	19.67	22.68	20.17	23.31	53	23.50	26.86	25.97	29.49
11	15.11	18.73	15.85	19.64	54	16.23	20.09	19.58	22.79
12	26.22	29.43	26.05	29.08	55	22.64	25.97	24.89	28.26
13	18.51	21.38	19.82	23.18	56	19.58	22.56	19.84	23.50
14	24.83	27.85	25.49	28.75	57	27.83	31.63	28.80	32.23
**15**[#]	**30.43**	33.70	**34.69**	UD	58	17.95	21.36	18.97	22.41
16	16.80	20.27	17.59	20.94	59	20.27	23.96	22.46	25.85
17	19.24	22.37	22.93	26.01	60	27.96	30.91	28.07	31.55
18	17.78	20.88	20.24	23.35	61	21.32	24.56	23.35	26.51
19	15.81	19.12	18.24	21.73	62	23.60	26.99	25.80	28.79
20	21.47	24.63	23.89	27.12	63	21.24	23.89	21.04	23.84
21	26.63	30.24	28.54	31.21	64	15.34	18.64	19.69	23.32
**22**[*]	**31.36**	32.83	**33.73**	UD	65	15.50	18.82	17.52	20.89
23	15.89	19.18	18.74	21.83	66	24.55	27.87	27.63	31.93
**24**[#]	**29.88**	33.71	**31.92**	35.56	67	17.04	20.42	19.63	22.84
25	17.31	20.75	19.94	22.96	68	23.79	27.53	25.80	29.46

26	19.15	22.68	21.53	24.79	69	21.87	25.19	22.17	25.39
27	19.20	20.23	22.53	23.53	70	16.79	20.21	18.09	21.07
28	13.48	14.86	16.51	18.14	71	27.79	29.53	27.98	31.20
**29**#	**27.39**	27.95	**30.70**	32.12	72	17.75	21.77	18.92	22.16
30	21.35	22.35	24.94	25.04	73	16.29	19.78	17.39	20.68
31	20.04	22.31	23.38	25.80	74	18.20	21.57	20.05	23.18
**32***	**31.82**	31.27	**31.88**	32.55	75	27.66	30.70	27.06	30.11
**33***	**UD**	31.18	**32.25**	30.65	76	17.06	19.66	19.03	22.24
34	21.81	25.11	24.80	27.86	77	16.11	19.23	20.86	24.07
35	23.32	26.76	26.78	30.74	78	22.09	24.98	23.50	26.25
36	19.29	22.59	18.33	21.79	79	15.29	18.63	16.12	19.32
37	22.86	26.46	23.89	27.79	80	22.56	25.61	24.10	27.17
38	19.82	22.86	20.23	23.71	81	17.79	20.78	24.68	28.75
39	12.21	15.66	14.32	17.43	82	18.96	22.10	21.45	24.94
40	21.03	24.36	24.72	27.79	83	21.85	24.84	23.17	25.59
41	14.71	NA	14.87	NA	84	24.77	27.81	29.54	32.18
42	17.98	NA	20.61	NA	85	21.60	24.93	23.97	27.46
43	21.84	NA	23.98	NA					

All stools initially tested positive for Salmonella spp. using BD MAX™ and serotypes have been isolated. UD: undetected; NA: Data not available; Ct: cycle threshold; qPCR: real-time PCR; vPCR: viability PCR. All stools were either semi-solid or liquid and fell under type 6 and 7 of the Bristol Stool Form Scale. * Stools that tested qPCR and vPCR negative (n=4). # Stools that tested qPCR positive and vPCR negative (n=5).

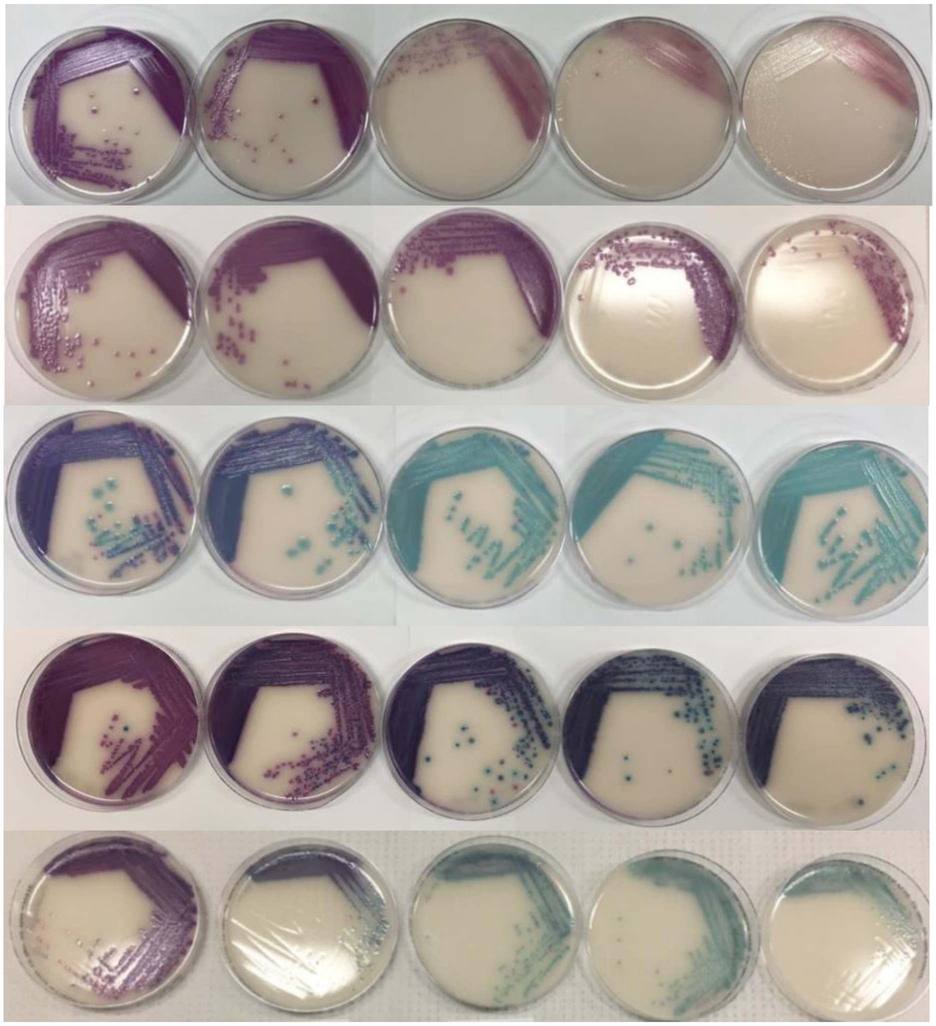

Bacterial cell dilution	Neat	-1	-2	-3	-4
Bacterial cell count (CFU/ 10 µL)	10^7	10^6	10^5	10^4	10^3

Supplementary Figure S1: Quadrant growth of five replicates from the stool broth standard curves.
